# Diagnostic value of ultrasonography in acute lateral and syndesmotic ligamentous ankle injuries

**DOI:** 10.1007/s00330-020-07305-7

**Published:** 2020-10-07

**Authors:** Thomas P. A. Baltes, Javier Arnáiz, Liesel Geertsema, Celeste Geertsema, Pieter D’Hooghe, Gino M. M. J. Kerkhoffs, Johannes L. Tol

**Affiliations:** 1 Research Department, Aspetar Orthopaedic and Sports Medicine Hospital, Sports City Street 1, P.O. Box 29222, Doha, Qatar; 2 Department of Orthopaedic Surgery, Amsterdam UMC, University of Amsterdam, Amsterdam Movement Sciences, Amsterdam, The Netherlands; 3 Academic Center for Evidence Based Sports Medicine (ACES), Amsterdam, The Netherlands; 4 Amsterdam Collaboration for Health and Safety in Sports (ACHSS), AMC/VUmc IOC Research Center, Amsterdam, The Netherlands; 5 Department of Radiology, Aspetar Orthopaedic and Sports Medicine Hospital, Doha, Qatar; 6 Department of Sports Medicine, Aspetar Orthopaedic and Sports Medicine Hospital, Doha, Qatar; 7 Department of Orthopaedic Surgery, Aspetar Orthopaedic and Sports Medicine Hospital, Doha, Qatar

**Keywords:** Ankle injuries, Ligaments, Ultrasound, Magnetic resonance imaging

## Abstract

**Objectives:**

To determine the diagnostic value of ultrasonography for complete discontinuity of the anterior talofibular ligament (ATFL), the calcaneofibular ligament (CFL) and the anterior inferior tibiofibular ligament (AITFL).

**Methods:**

All acute ankle injuries in adult athletes (> 18 years old) presenting to the outpatient department of a specialised Orthopaedic and Sports Medicine Hospital within 7 days post-injury were assessed for eligibility. Using ultrasonography, one musculoskeletal radiologist assessed the ATFL, CFL and AITFL for complete discontinuity. Dynamic ultrasound measurements of the tibiofibular distance (mm) in both ankles (injured and contralateral) were acquired in the neutral position (N), during maximal external rotation (Max ER), and maximal internal rotation (Max IR). MR imaging was used as a reference standard.

**Results:**

Between October 2017 and July 2019, 92 acute ankle injuries were included. Ultrasound diagnosed complete discontinuity of the ATFL with 87% (CI 74–95%) sensitivity and 69% (CI 53–82%) specificity. Discontinuity of the CFL was diagnosed with 29% (CI 10–56%) sensitivity and 92% (CI 83–97%) specificity. Ultrasound diagnosed discontinuity of the AITFL with 100% (CI 74–100%) sensitivity and 100% (CI 95–100%) specificity. Of the dynamic measurements, the side-to-side difference in external rotation had the highest diagnostic value for complete discontinuity of the AITFL (sensitivity 82%, specificity 86%; cut-off 0.93 mm).

**Conclusions:**

Ultrasound has a good to excellent diagnostic value for complete discontinuity of the ATFL and AITFL. Therefore, ultrasound can be used to screen for injury of the ATFL and AITFL. Compared with ultrasound, dynamic ultrasound has inferior diagnostic value for complete discontinuity of the AITFL.

**Key Points:**

*• Ultrasound has a good to excellent diagnostic value for complete discontinuity of the anterior talofibular ligament (ATFL) and anterior inferior tibiofibular ligament (AITFL).*

*• Ultrasound can be used to screen for injury of the ATFL and AITFL.*

*• Compared with ultrasound, dynamic ultrasound has inferior diagnostic value for complete discontinuity of the AITFL.*

**Electronic supplementary material:**

The online version of this article (10.1007/s00330-020-07305-7) contains supplementary material, which is available to authorized users.

## Introduction

Acute ligamentous ankle injuries are one of the most common injuries in sports [1]. Depending on the trauma mechanism, acute ankle sprains may injure the lateral ankle ligaments and/or syndesmotic and/or the deltoid ligaments [2]. As physical examination in the acute phase (days 1–5) has proven to be of limited diagnostic accuracy, magnetic resonance imaging (MRI) is increasingly used in athletes [3–6]. Ultrasound has the potential to provide an inexpensive and easily accessible alternative that could be used to screen for ligamentous ankle injuries [7].

The diagnostic value of ultrasound for acute injury of the anterior talofibular ligament (ATFL) has been investigated in various studies [8]. However, the diagnostic value of a systematic approach (including the calcaneofibular ligament (CFL) and syndesmosis) to acute ligamentous ankle injuries has only been reported in two studies [9, 10]. The main limitation in these studies is that the diagnostic values were reported for ligamentous injury, without the differentiation between partial and complete tears. As only complete tears are considered amenable to surgical repair, a study investigating the diagnostic value of ultrasound for complete ligamentous discontinuity is warranted [11, 12].

Dynamic ultrasound has been reported as an accurate method of diagnosing syndesmosis injury [13]. However, no prospective cohort study in an unselected cohort of athletes has been performed. Therefore, a study validating the diagnostic accuracy of dynamic ultrasound in a large prospective cohort of athletes with acute ankle injuries is necessary.

The aim of this study was to establish the diagnostic value of ultrasound for complete discontinuity of the lateral ankle ligaments and AITFL in athletes with an acute ankle injury. Our secondary aim was to establish the diagnostic value of dynamic ultrasound for complete discontinuity of the AITFL. Our hypothesis is that in athletes with an acute ankle injury, (dynamic-) ultrasound has excellent diagnostic accuracy for complete discontinuity of the ATFL, CFL and AITFL.

## Methods

### Patient selection

Between October 2017 and July 2019, all patients presenting to the outpatient department of a specialised Orthopaedic and Sports Medicine Hospital within 7 days after an acute ankle injury were asked to participate in this study. Inclusion criteria were as follows: acute ankle injuries in adult athletes (≥ 18 years old), participating in sports at a professional or recreational level and presenting within 7 days of injury. Ankle injuries were excluded if imaging studies demonstrated an ankle fracture or if the ultrasound and MRI studies could not be acquired within 10 days post-injury. Ethical approval was acquired from the Anti-Doping Lab Qatar review board (IRB No. F2016000153). Written informed consent was obtained from all patients at the time of inclusion.

### Power calculation

This study was part of a large prospective cohort study on the functional outcome and return to play of acute ligamentous ankle injuries. The sample size estimations were therefore based on an expected difference in functional outcome. No a priori sample size calculation was performed for the present study.

### Ultrasound imaging

**Fig. 1:**
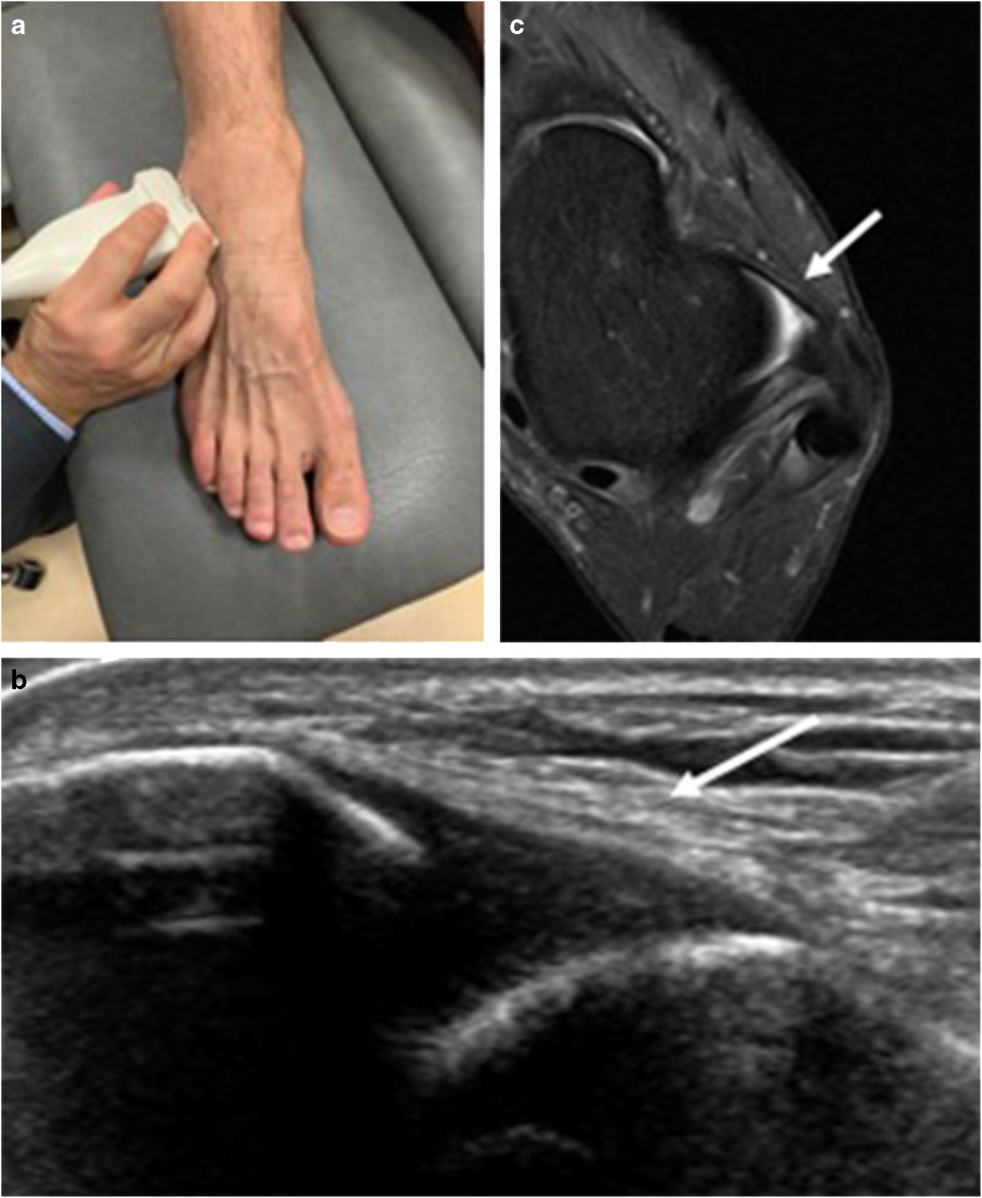
Probe position and (US/MR) imaging results for the anterior talofibular ligament (ATFL). Ultrasound for the diagnosis of anterior talofibular ligament (ATFL) injury. **a** The ultrasound probe is placed in the transverse plane (longitudinal to the ATFL) anterior to the tip of the lateral malleolus. **b** Ultrasound findings consistent with an intact anterior talofibular ligament (arrow). **c** Axial PD-FS image showing an intact anterior tibiofibular ligament (arrow)

**Fig. 2:**
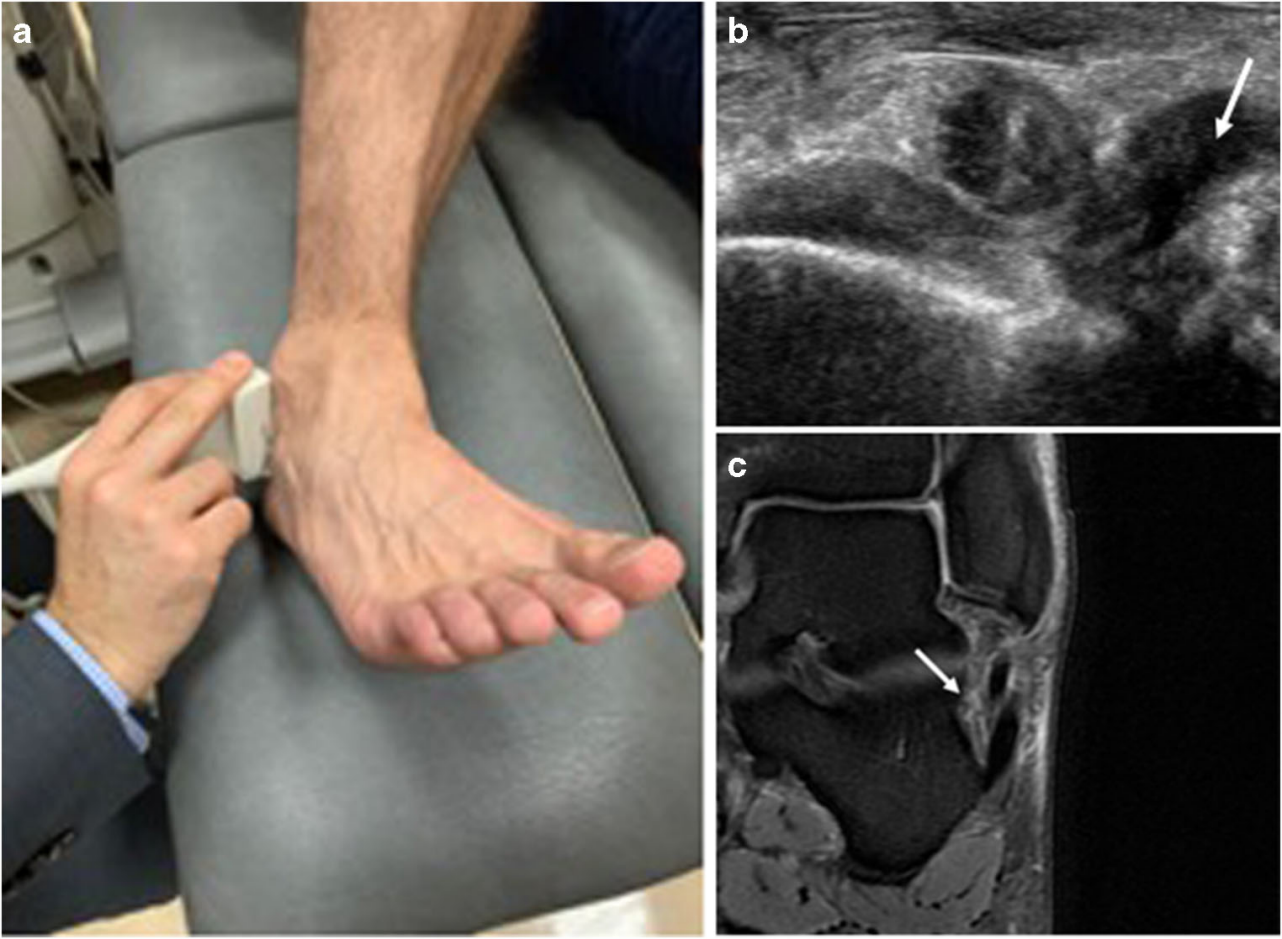
Probe position and (US/MRI) imaging results for the calcaneofibular ligament (CFL). Ultrasound for the diagnosis of calcaneofibular ligament (CFL) injury. **a** The ultrasound probe is placed in the frontal plane (longitudinal to the calcaneofibular ligament). **b** Ultrasound findings consistent with a complete tear of the calcaneofibular ligament (arrow). **c** Axial PD-FS image showing waviness of the calcaneofibular ligament consistent with a complete tear (arrow)

**Fig. 3:**
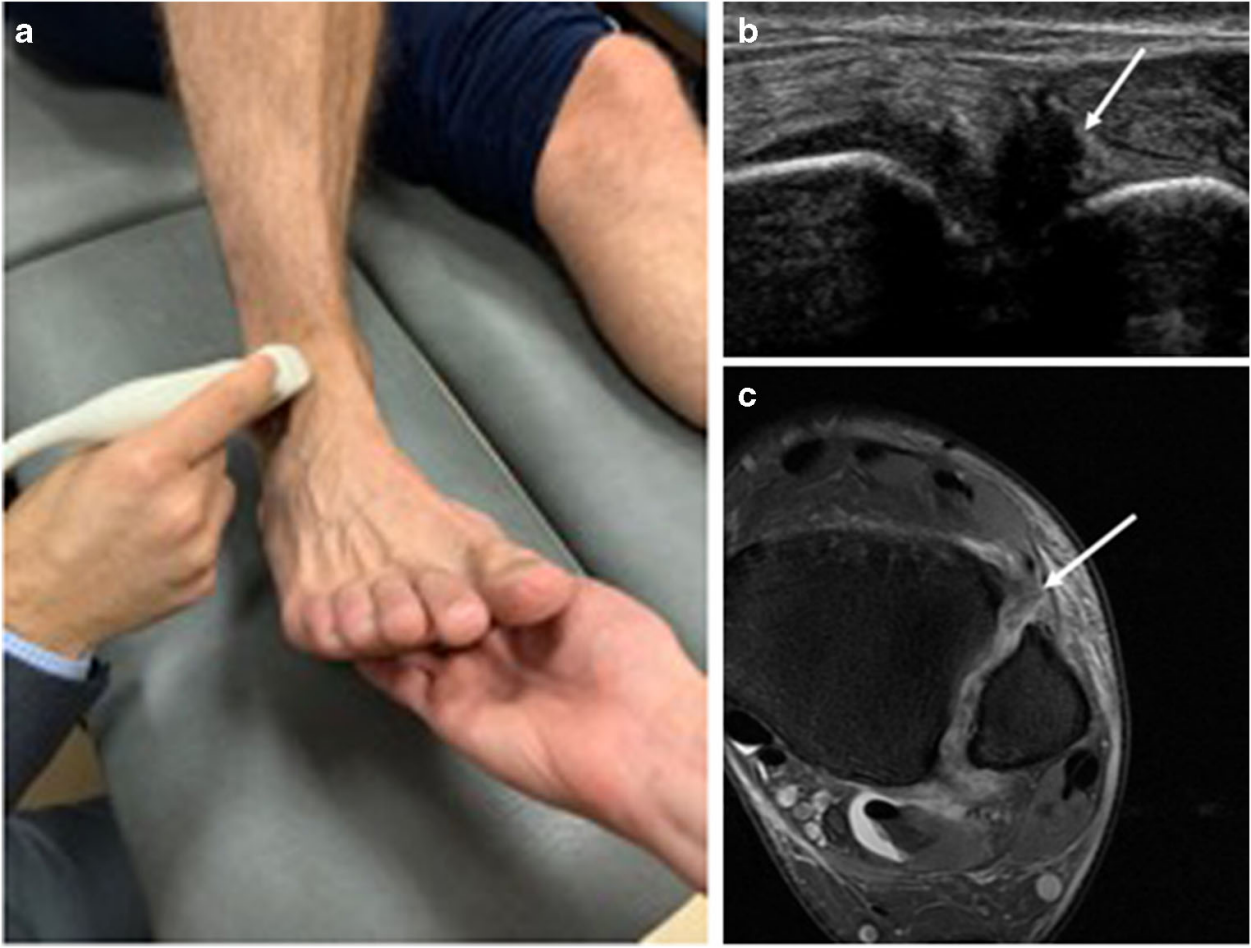
Probe position and (US/MR) imaging findings for the anterior tibiofibular ligament (AITFL). Ultrasound for the diagnosis of anterior tibiofibular ligament injury. **a** The ultrasound probe is placed over the AITFL in the axial plane (about 1 cm proximal to the joint line). **b** Ultrasound findings consistent with a complete tear of the anterior tibiofibular ligament (arrow). **c** Axial PD-FS image showing disruption of the anterior tibiofibular ligament, consistent with a complete tear (arrow)

All examinations were performed by the same MSK radiologist (J.A.), with 13 years of experience in MSK ultrasound. An ultrasound device (iU22, Philips) with a high-frequency linear transducer (5–12 MHz) was used for standardised sonographic evaluation. Patients were examined in a supine position with their knees in 90° flexion. Ultrasound of the ATFL was performed by placing the transducer in the transverse plane (longitudinal to the ATFL) anterior to the tip of the lateral malleolus (Fig. 1a). The CFL was visualised with the probe in the frontal plane (longitudinal to the CFL) (Fig. 2a). For visualisation of the anterior tibiofibular ligament, the transducer was placed over the AITFL in the axial plane, about 1 cm proximal to the joint line (Fig. 3a). During the examination of the AITFL, an assistant provided 5–10° passive dorsal flexion of the ankle.

**Fig. 4:**
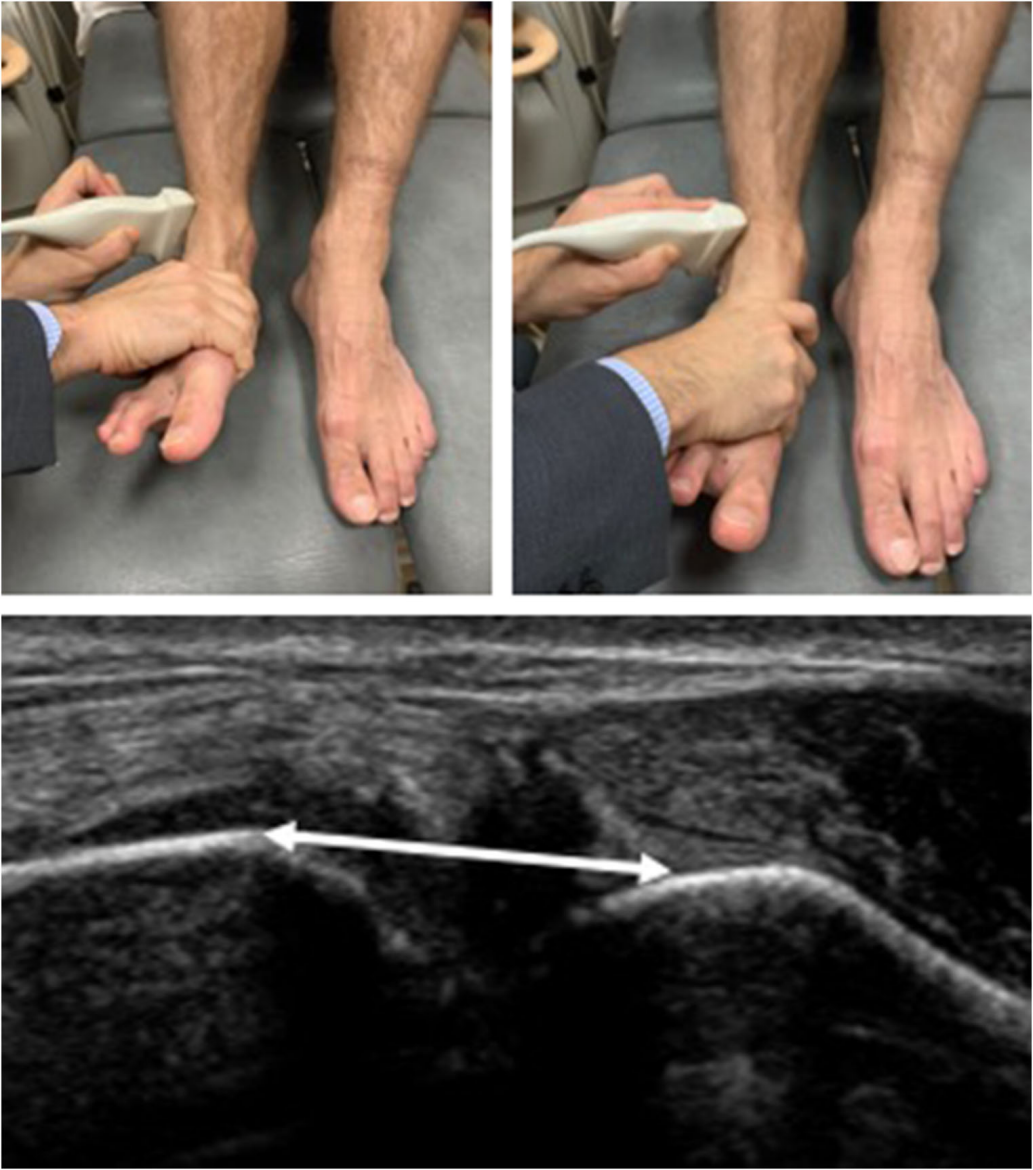
Probe position and manoeuvres for dynamic measurement of the tibiofibular clear space. Dynamic manoeuvres of the ankle. **a** Passive dorsal flexion and maximal external rotation manoeuvre of the ankle. **b** Passive dorsal flexion and internal rotation manoeuvre of the ankle. **c** Measurements of the tibiofibular clear space in mm (double-headed arrow)

After the initial sonographic examination, dynamic ultrasound measurements were obtained, as originally described by Mei-Dan et al [13]. With the knees kept together in 90° flexion, the foot of the injured ankle was passively brought into 5–10° dorsal flexion (Fig. 3a). The transducer was placed over the AITFL in the axial plane, about 1 cm proximal to the joint line. After the tibiofibular distance was measured (in mm) in the neutral position (N), maximal external rotation (Max ER) and maximal internal rotation (Max IR) manoeuvres were performed (Fig. 4a, 4b) The radiologist applied external rotation and internal rotation stress until a firm endpoint was reached [13]. During the stress manoeuvres, a digital video clip was recorded from which the tibiofibular distance was measured at the point of maximal external rotation and maximal internal rotation. The examination was repeated for the contralateral uninjured ankle.

### Magnetic resonance imaging

MRI was used as the reference standard, since MRI has demonstrated excellent diagnostic accuracy for injury of the lateral ankle ligaments and syndesmosis ligaments [14–16]. Patients underwent a wide-bore 3.0-T MRI (GE Discovery, GE Healthcare) using an 8-channel receive-only foot and ankle array (Invivo, Philips Healthcare). In the sagittal plane, T1-weighted (repetition time [TR] 400–680 ms; echo time [TE] 10–11 ms; 3.0-mm slice thickness; 0.5-mm interslice gap; 416 × 288 pixel matrix; 2 excitations [NEX] 16 cm^2^ field of view [FOV]; echo train length [ETL] 3) and proton density fat saturated [PD-FS] (TR 2500–3200 ms, TE 32–35 ms, 3.0-mm slice thickness, 0.5-mm interslice gap; 352 × 526 pixel matrix; 2 NEX; 20 cm^2^ FOV; ETL 8) sequences were obtained, axial T2-weighted (TR 5500–6700 ms; TE 72–80 ms; 3.5-mm slice thickness, 0.5-mm interslice gap; 320 × 224 pixel matrix; 2 NEX; 13 cm^2^ FOV; ETL 16) and PD-FS sequences (TR 2900–4000; TE 35–39 ms; 3.5-mm slice thickness, 0.5-mm interslice gap; 320 × 224 pixel matrix; 2 NEX; 13 cm^2^ FOV; ETL 6) were acquired and in the coronal plane, a PD-FS (TR 2700–3400; TE 35–38 ms; 3.5-mm slice thickness; 0.5-mm interslice gap; 320 × 224 pixel matrix; 2 NEX; 16 cm^2^ FOV; ETL 6) sequence was obtained.

### Outcome measurements

Blinded to clinical information, the same MSK radiologist (J.A.) graded the ultrasound and MR scans. The ultrasound scans were graded during the sonographic examination. To assure blinding of the radiologist to the results of the ultrasound scan, MR scans were graded after a period of minimal 28 days. Injury of the individual ligaments (ATFL; CFL, AITFL) were graded according to the Schneck grading system [17] (Supplementary Appendix 1; grade 0: normal; grade 1: peri-ligamentous high signal/edema on proton density–weighted sequences and no discontinuity of fibres; grade 2: partial discontinuity but preserved remnant fibres; grade 3: complete discontinuity).

The following individual ankle ligaments were graded according to the four grade grading system: the lateral ankle ligaments (anterior talofibular ligament [ATFL]; calcaneofibular ligament [CFL]) and one of the syndesmosis ligaments (anterior inferior tibiofibular ligament [AITFL])

### Dynamic measurements

The dynamic measurements included the tibiofibular distance (in mm) in the three positions: neutral (N); maximal internal rotation (Max IR); maximal external rotation (Max ER). From these measurements, two composite dynamic measurements were calculated. The first measurement (Δ ER-N) was calculated by subtracting the tibiofibular distance in the neutral position from the tibiofibular distance in maximal external rotation. The second composite measurement (Δ N-IR) was calculated by subtracting the tibiofibular distance in maximal internal rotation from the tibiofibular distance in the neutral position.

### Statistical analysis

To evaluate the diagnostic value of ultrasound for complete ligamentous discontinuity, the Schneck grading of the individual ligaments was dichotomised to (1) no complete discontinuity (grade 0: normal ligament, grade 1: peri-ligamentous edema and grade 2: partial discontinuity) or (2) complete discontinuity (grade 3: complete discontinuity). For an exploratory post hoc analysis of the diagnostic values for injury of the individual ankle ligaments, the Schneck grading was dichotomised as (1) no injury (grade 0 and grade 1) and (2) injury (grade 2 and grade 3).

The diagnostic value of the dynamic measurements to distinguishing both groups (group A: no complete discontinuity AITFL vs. group B: complete discontinuity AITFL) was established in two comparisons. For the first comparison, the tibiofibular clear space was compared between group A (no complete discontinuity AITFL) and group B (complete discontinuity AITFL). For the second comparison, the side-to-side differences in tibiofibular distance between the injured and contralateral (uninjured) ankle were compared between group A (no complete discontinuity AITFL) and group B (complete discontinuity AITFL)

Descriptive statistics was used to present patient demographics and number of ligamentous lesions observed. Categorical data was presented as frequencies with percentages; continuous variables were presented as mean with standard deviation (SD) for data with a normal distribution and as median with interquartile range (IQR) in case of non-normal distribution. Data distribution was assessed using the Shapiro-Wilk test and visual inspection.

The diagnostic value of ultrasound for complete ligamentous discontinuity including sensitivity, specificity, positive predictive value (PPV), negative predictive value (NPV) and positive and negative likelihood ratios (LR+ and LR-) that were calculated using a 2 × 2 table.

The diagnostic value and optimal cut-off (Youden’s index = maximal value) for each dynamic measurement were calculated using a ROC curve. If a dynamic measurement was missing, the patient was excluded from the analysis of the specific dynamic measurement. An independent *t* test was used to compare groups for each dynamic measurement. Significance was set at *p* < 0.05. Statistical analysis was performed using the Rstudio (Rstudio V 1.2.1335).

## Results

### Baseline characteristics

**Fig. 5:**
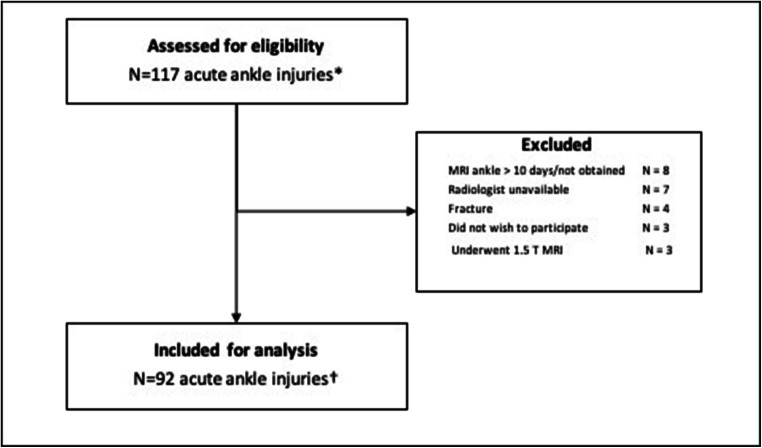
Flowchart. In 116 athletes (asterisk); In 91 athletes (dagger)

Between October 2017 and July 2019, a total of 117 acute ankle injuries were assessed for eligibility (Fig. 5) Ninety-two acute ankle injuries were included in this study, of which one was a subsequent contralateral ankle injury. The median age at the time of injury was 25 years (IQR 8), with a range of 18 to 45 years. The majority of included patients were male (92%). The median time from injury to ultrasound was 2 days (IQR 3). The MR scans were acquired with a median of 3 days (IQR 3) post-injury. Of the 91 included patients, 50 (55%) played football/futsal, 18 (20%) volleyball, 8 (9%) handball, 6 (7%) basketball and 9 (10%) participated in other sports.

### Grading of individual ligaments

The prevalence and diagnostic values (sensitivity/specificity, likelihood ratios, negative/positive predictive value) per individual ligament are detailed in Table 1. Grading of the individual ligaments prior to dichotomisation is provided in Supplementary Appendix 2. On the reference standard (MRI), complete discontinuity of the ATFL was observed in 47 (51%) and for the CFL in 17 (18%) acute ankle injuries. For the lateral ankle ligaments, the diagnostic value of ultrasound for complete discontinuity of the ATFL was 87% (CI 74–95%) for sensitivity and 69% (CI 53–82%) for specificity. Complete discontinuity of the CFL was diagnosed with 29% (CI 10–56%) sensitivity and 92% (CI 83–97%) specificity. For the syndesmosis, complete discontinuity of the AITFL was present in 12 (13%) out of 92 included acute ankle injuries. Ultrasound diagnosed complete discontinuity of the AITFL with 100% (CI 74–100%) sensitivity and 100% (CI 95–100%) specificity. The analysis for the diagnostic values for injury (defined as partial or complete discontinuity) of the individual ligaments is provided in Table 2.

**Table 1:** The prevalence and diagnostic values of ultrasonography for complete discontinuity of the individual ankle ligaments in 92 athletes presenting with an acute ankle injury. MRI was used as a reference standard

	Prevalence (MRI)	Prevalence (US)	Sensitivity	Specificity	LR+	LR-	PPV	NPV
Lateral ankle ligaments								
ATFL	47/92 (0.51)	55/92 (0.60)	0.87 (0.74–0.95)	0.69 (0.53–0.82)	2.80 (1.79–4.39)	0.19 (0.09–0.40)	0.75 (0.61–0.85)	0.84 (0.68–0.94)
CFL	17/92 (0.18)	11/92 (0.12)	0.29 (0.10–0.56)	0.92 (0.83–0.97)	3.68 (1.27–10.65)	0.77 (0.56–1.05)	0.45 (0.17–0.77)	0.85 (0.76–0.92)
Syndesmosis ligaments								
AITFL	12/92 (0.13)	12/92 (0.13)	1.00 (0.74–1.00)	1.00 (0.95–1.00)	Infinity (N/a–Inf.)	0.00 (0.00–N/a)	1.00 (0.74–1.00)	1.00 (0.95–1.00)

All diagnostic values are presented with 95% confidence interval (95%CI); *ATFL*, anterior talofibular ligament; *CFL*, calcaneofibular ligament; *AITFL*, anterior inferior tibiofibular ligament; *MRI*, magnetic resonance imaging; *US*, ultrasonography; *LR+*, positive likelihood ratio; *LR-*, negative likelihood ratio; *PPV*, positive predictive value; *NPV*, negative predictive value; *N/a*, not applicable; *Inf.*, infinity

**Table 2:** The prevalence and diagnostic values of ultrasonography for partial and complete discontinuity of the individual ankle ligaments in 92 athletes presenting with an acute ankle injury. MRI was used as a reference standard

	Prevalence (MRI)	Prevalence (US)	Sensitivity	Specificity	LR+	LR-	PPV	NPV
Lateral ankle ligaments								
ATFL	61/92 (0.66)	58/92 (0.63)	0.89 (0.77–0.95)	0.87 (0.69–0.96)	6.86 (2.74–17.20)	0.13 (0.07–0.27)	0.93 (0.82–0.98)	0.79 (0.62–0.91)
CFL	45/92 (0.49)	26/92 (0.28)	0.49 (0.34-0.64)	0.91 (0.79–0.97)	5.74 (2.15–15.36)	0.56 (0.42–0.75)	0.85 (0.64–0.95)	0.65 (0.52–0.76)
Syndesmosis ligaments								
AITFL	14/92 (0.15)	12/92 (0.13)	0.86 (0.56-0.97)	1.00 (0.94–1.00)	Infinity (N/a–Inf.)	0.14 (0.04–0.52)	1.00 (0.70–1.00)	0.98 (0.90–1.00)

All diagnostic values are presented with 95% confidence interval (95%CI); *ATFL*, anterior talofibular ligament; *CFL*, calcaneofibular ligament; *AITFL*, anterior inferior tibiofibular ligament; *MRI*, magnetic resonance imaging; *US*, ultrasonography; *LR+*, positive likelihood ratio; *LR-*, negative likelihood ratio; *PPV*, positive predictive value; *NPV*, negative predictive value; *N/a*, not applicable; *Inf.*, infinity

### Dynamic measurements (tibiofibular distance)

The mean tibiofibular distance per dynamic measurement (N, Max IR, Max ER, Δ ER-N and Δ N-IR) and the corresponding diagnostic values (AUC, cut-off point, sensitivity/specificity) are detailed in Table 3. Due to discomfort, dynamic measurements were only acquired in 89 out of 92 acute ankle injuries (internal rotation in 88). The mean tibiofibular distance in the neutral position (10.08 mm vs. 11.32 mm, *p* = 0.02) and in the maximal external rotation position (10.13 mm vs. 11.75 mm, *p* = 0.002) was higher for the group with a discontinuous AITFL. The sensitivity and specificity of these dynamic measurements were 58% and 79–90%, respectively. The other dynamic measurements (IR, Δ ER-N, Δ N-IR) were not significantly different between groups.

**Table 3:** Mean tibiofibular distance (in mm, ± SD) per dynamic manoeuvre in *N* acute ankle injuries; Comparison 1: mean tibiofibular distance in group A (no complete discontinuity of AITFL) versus group B (complete discontinuity of the AITFL); Comparison 2: the mean difference between tibiofibular distance in the injured ankle and the contralateral (uninjured) ankle, compared between group A and group B

	N (group A vs. group B)	TFD group A	TFD group B	*p* value	AUC (95%CI)	Cut-off	Sensitivity	Specificity
Comparison: tibiofibular distance								
N	89 (77 vs. 12)	10.1 (± 1.6)	11.3 (± 2.3)	*p* = 0.018	0.65 (0.47–0.84)	11.3	0.58	0.79
IR	88 (76 vs. 12)	10.1 (± 1.6)	11.1 (± 2.1)	*p* = 0.054	0.65 (0.44–0.85)	10.6	0.67	0.72
ER	89 (77 vs. 12)	10.1 (± 1.6)	11.8 (± 2.1)	*p* = 0.002	0.72 (0.54–0.90)	11.9	0.58	0.90
Δ ER-N	89 (77 vs. 12)	0.1 (± 0.7)	0.4 (± 1.2)	*p* = 0.132	0.60 (0.40–0.80)	0.3	0.58	0.66
Δ N-IR	88 (76 vs. 12)	0.0 (± 0.9)	0.3 (± 1.2)	*p* = 0.412	0.58 (0.34–0.81)	0.6	0.58	0.83
Comparison: side-to-side difference tibiofibular distance								
N	83 (72 vs. 11)	0.2 (± 1.3)	2.04 (± 1.8)	*p* < 0.001	0.79 (0.65–0.94)	1.2	0.73	0.76
IR	81 (70 vs. 11)	0.0 (± 1.4)	1.71 (± 1.9)	*p* < 0.001	0.77 (0.59–0.96)	1.4	0.73	0.83
ER	83 (72 vs. 11)	0.0 (± 1.2)	1.89 (± 1.4)	*p* < 0.001	0.88 (0.79–0.98)	0.9	0.82	0.86
Δ ER-N	83 (72 vs. 11)	0.2 (± 1.1)	- 0.15 (± 1.1)	*p* = 0.824	0.48 (0.29–0.68)	- 0.4	0.58	0.64
Δ N-IR	81 (70 vs. 11)	0.2 (± 1.3)	0.32 (± 1.6)	*p* = 0.71	0.50 (0.28–0.71)	-0.7	0.80	0.36

Group A: all acute ankle injuries with no complete discontinuity of the AITFL per MR scan; Group B: all acute ankle injuries with complete discontinuity of the AITFL per MR scan. AUC values are presented with corresponding 95% confidence interval (95%CI); *N*, neutral; *Max IR*, maximal internal rotation; *Max ER*, maximal external rotation; *ΔER-N*, maximal external rotation-neutral; *ΔN-IR*, neutral-maximal internal rotation; *N*, number of included measurements; *TFD*, tibiofibular distance; *AUC*, area under the curve; cut-off at Youden’s index = maximal value (in mm)

### Dynamic measurements (side-to-side difference)

The mean side-to-side difference per dynamic measurement (N, Max IR, Max ER, Δ ER-N and Δ N-IR) and the corresponding diagnostic values (AUC, cut-off point, sensitivity/specificity) are detailed in Table 3. Dynamic measurements in the contralateral ankle were performed in 83 ankles (internal rotation in 81). The mean side-to-side difference in tibiofibular distance in the neutral position (0.20 mm vs. 2.04 mm, *p* < 0.001), during maximal internal rotation (0.04 mm vs. 1.71 mm, *p* < 0.001) and during maximal external rotation (- 0.03 mm vs 1.89 mm, *p* < 0.001) was higher for the group with complete discontinuity of the AITFL. The highest AUC was observed for the side-to-side difference in maximal external rotation (AUC 0.88; sensitivity 82%, specificity 86% at cut-off 0.93 mm). The side-to-side difference for the other dynamic measurements (Δ ER-N, Δ N-IR) was not significantly different between groups and resulted in poor diagnostic values.

## Discussion

The most important finding of this study is that ultrasonography resulted in good to excellent diagnostic values for complete discontinuity of the ATFL and AITFL compared with the reference standard of MR imaging. In contrast to our hypothesis, ultrasound had poor sensitivity for complete discontinuity of the CFL. Based on these findings, ultrasonography can only be used to screen for the presence of complete discontinuity of the ATFL and the AITFL. For dynamic ultrasound, the side-to-side difference in the tibiofibular distance during maximal external rotation had the highest diagnostic accuracy. However, the included dynamic measurements had inferior diagnostic accuracy compared with plain ultrasonography of the AITFL.

### Diagnostic value of US for the individual ligaments

The diagnostic value of ultrasound in the diagnosis of acute injury of the ATFL has been investigated in various studies, primarily by comparing ultrasound with surgical findings as a reference standard [8]. However, the methodological quality of these studies is limited as surgical exploration in most studies was restricted to those patients with positive imaging findings only. Therefore, establishing the diagnostic value of ultrasound with MRI as a reference is a valuable alternative. So far, two studies have compared the diagnostic value of ultrasound for injury of the lateral ankle ligaments against MRI [18, 19]. In the first study by Margetic et al in 30 patients with an acute ankle sprain, ultrasound had a sensitivity of 60% and specificity of 100% for complete tears of the ATFL. Tears of the CFL were diagnosed with 0% sensitivity and 100% specificity [18]. Although the methodology is similar to our study, the reported specificity was higher and sensitivity lower for both lateral ankle ligaments. This can be contributed to the low number of disease-positive cases (complete tear) in their study, making the findings less robust. In a more recent study by Gun C et al, 65 patients underwent bedside ultrasound, followed by MRI. In this study, ultrasound had 93.8% sensitivity and 100% specificity for injury of the ATFL. However, no grading of injury severity was applied, hindering the comparison of outcomes [19].

The systematic ultrasound approach to the diagnosis of ligamentous ankle injuries has been investigated in two studies [7, 8]. In a prospective study by Milz et al, 64 patients with an acute ankle injury underwent ultrasound followed by a 0.2-T MR scan. In this study, ultrasound had a sensitivity of 98% and a specificity of 83% for injury (complete and partial tears) of the ATFL. Injury of the CFL was diagnosed with 87% sensitivity and 89% specificity [8]. In a more recent study by Lee et al, injury (complete and partial tears) of the ATFL was diagnosed with 99–100% sensitivity and 95% specificity. Injury of the CFL was diagnosed with 96–100% sensitivity and 97–100% specificity. Compared with MR imaging, ultrasound established injury of the AITFL with a 100% sensitivity and 100% specificity [7]. The superior diagnostic values for the ATFL and CFL in the study by Lee et al might be explained by the fact that in our study, differentiation of partial tears from complete tears was applied.

### Diagnostic value of dynamic US measurements

Only one previous study has reported on the diagnostic value of dynamic ultrasound measurements in the diagnosis of syndesmosis injury [13]. In this study, Mei-Dan et al compared dynamic tibiofibular clear space measurements in 9 patients with a recent syndesmotic injury to a control group of 20 patients clinically diagnosed with a lateral ankle sprain and 18 uninjured control subjects. They found the side-to-side difference in tibiofibular clear space between those with a syndesmosis injury and the control group, to be statistically significant in all positions (N, IR, ER). The diagnostic accuracy for the side-to-side difference in the N and ER positions was 100% at a cut-off of 0.7 mm and 0.9 mm, respectively. Our study confirmed the side-to-side difference in external rotation as the superior dynamic measurement. However, we obtained inferior diagnostic values compared with the study by Mei-Dan et al. This can potentially be explained by the fact that in our study, an unselected cohort of athletes was included.

In comparison with the study by Mei-Dan et al, the mean tibiofibular distance in both groups (injured and uninjured) of our study was higher. A possible explanation for this discrepancy is that we might have measured the tibiofibular distance more superficially, towards the posterior border of the AITFL. Alternatively, anatomical variations between patient populations might have contributed to the difference in the findings.

Few other studies have looked at dynamic measurements of the tibiofibular distance, but none of these studies reported diagnostic values (sensitivity/specificity) for the individual dynamic tibiofibular clear space measurements [20–22].

### Strength and limitations

This is the first study to prospectively compare the diagnostic values of (dynamic-) ultrasound in an athletic cohort of acute ankle injuries. However, minor shortcomings are present in this study. In this study, we used a 5–1-MHz probe. In theory, a high-frequency probe (5–18 MHz) could have yielded more accurate results. However, as we strived to provide an outcome that could easily be translated into clinical practice, we investigated the most commonly used 5–12-Mhz probe [23]. Secondarily, for the dynamic manoeuvres, we used unstandardised dynamic measurements as this was most applicable to the clinical situation [13]. Future research investigating standardised (instrumented) dynamic measurements may be able to improve the diagnostic accuracy and aid in the diagnosis of syndesmotic instability. However, clinical applicability should be an important element in such research. In addition, due to the low prevalence of deltoid lesions in acute ankle injuries, the diagnostic value of ultrasound for the diagnosis of deltoid injury was not evaluated. Furthermore, all ultrasounds were performed and graded by a single senior MSK radiologist with extensive experience in MSK sonography. As the inter-rater reliability of ultrasound for ligamentous ankle injuries was not established in this study, inferior results might be expected in less-experienced sonographers.

### Implications for clinical practice

As injury of the ATFL can be diagnosed accurately by delayed physical examination, the need for ultrasound evaluation of lateral ankle sprains is limited [5]. However, when suspecting a syndesmosis injury, ultrasound may be used to detect complete discontinuity of the AITFL. In acute ankle sprains involving the syndesmosis, the AITFL is the first ligament to be injured [3]. Therefore, ultrasound can be used to differentiate acute ankle sprains affecting the lateral ankle ligaments from those with an increased risk of syndesmotic instability. When an injury of the AITFL is detected with ultrasound imaging, syndesmotic stability can be further evaluated by MRI or arthroscopy [24]. Although a useful addition to the ultrasonographic evaluation of ligamentous ankle injuries, dynamic ultrasound should not be used as the sole method of diagnosing syndesmosis injury.

### Conclusion

Ultrasound has a good to excellent diagnostic value for the presence of complete discontinuity of the ATFL and AITFL. Therefore, ultrasound can be used to detect complete discontinuity of the ATFL and the anterior ligament of the ankle syndesmosis (AITFL). Compared with ultrasound, dynamic ultrasound has inferior diagnostic value for the diagnosis of complete discontinuity of the AITFL.

## Electronic supplementary material

ESM 1 (DOCX 2556 kb)

## Abbreviations

AITFL: Anterior inferior tibiofibular ligament
ATFL: Anterior talofibular ligament
CFL: Calcaneofibular ligament
IQR: Interquartile range
LR +: Positive likelihood ratios
LR-: Negative likelihood ratios
Max ER: Maximal external rotation
Max IR: Maximal internal rotation
MRI: Magnetic resonance imaging
MSK: Musculoskeletal
N: Neutral
NPV: Negative predictive value
PPV: Positive predictive value
SD: Standard deviation
US: ultrasound/ultrasonography

## References

[1] Fong DT-P, Hong Y, Chan L-K, Yung PS-H, Chan K-M (2007). A systematic review on ankle injury and ankle sprain in sports. Sports Med.

[2] Doherty C, Delahunt E, Caulfield B, Hertel J, Ryan J, Bleakley C (2014). The incidence and prevalence of ankle sprain injury: a systematic review and meta-analysis of prospective epidemiological studies. Sports Med.

[3] Großterlinden LG, Hartel M, Yamamura J (2016). Isolated syndesmotic injuries in acute ankle sprains: diagnostic significance of clinical examination and MRI. Knee Surg Sport Traumatol Arthrosc.

[4] Sman AD, Hiller CE, Rae K (2015). Diagnostic accuracy of clinical tests for ankle syndesmosis injury. Br J Sports Med.

[5] van Dijk CN, Mol BW, Lim LS, Marti RK, Bossuyt PM (1996). Diagnosis of ligament rupture of the ankle joint. Physical examination, arthrography, stress radiography and sonography compared in 160 patients after inversion trauma. Acta Orthop Scand.

[6] Crema MD, Krivokapic B, Guermazi A (2019). MRI of ankle sprain: the association between joint effusion and structural injury severity in a large cohort of athletes. Eur Radiol.

[7] Sconfienza LM, Albano D, Allen G (2018). Clinical indications for musculoskeletal ultrasound updated in 2017 by European Society of Musculoskeletal Radiology (ESSR) consensus. Eur Radiol.

[8] Lee SH, Yun SJ (2019) Ankle ultrasound for detecting anterior talofibular ligament tear using operative finding as reference standard: a systematic review and meta-analysis. Eur J Trauma Emerg Surg. 10.1007/s00068-019-01169-3 10.1007/s00068-019-01169-3 31187159

[9] Lee SH, Yun SJ (2017). The feasibility of point-of-care ankle ultrasound examination in patients with recurrent ankle sprain and chronic ankle instability: comparison with magnetic resonance imaging. Injury.

[10] Milz P, Milz S, Steinborn M, Mittlmeier T, Reiser M (1999). 13-MHc high frequency ultrasound of the lateral ligaments of the ankle joint and the anterior tibia-fibular ligament. Comparison and results of MRI in 64 patients. Radiologe.

[11] Vuurberg G, Hoorntje A, Wink LM (2018). Diagnosis, treatment and prevention of ankle sprains: update of an evidence-based clinical guideline. Br J Sports Med.

[12] McCollum GA, van den Bekerom MPJ, Kerkhoffs GMMJ, Calder JDF, van Dijk CN (2013). Syndesmosis and deltoid ligament injuries in the athlete. Knee Surg Sports Traumatol Arthrosc.

[13] Mei-Dan O, Kots E, Barchilon V, Massarwe S, Nyska M, Mann G (2009). A dynamic ultrasound examination for the diagnosis of ankle syndesmotic injury in professional athletes: a preliminary study. Am J Sports Med.

[14] Oae K, Takao M, Naito K (2003). Injury of the tibiofibular syndesmosis: value of MR imaging for diagnosis. Radiology.

[15] Takao M, Ochi M, Oae K, Naito K, Uchio Y (2003). Diagnosis of a tear of the tibiofibular syndesmosis. The role of arthroscopy of the ankle. J Bone Joint Surg Br.

[16] Oae K, Takao M, Uchio Y, Ochi M (2010). Evaluation of anterior talofibular ligament injury with stress radiography, ultrasonography and MR imaging. Skeletal Radiol.

[17] Schneck CD, Mesgarzadeh M, Bonakdarpour A (1992). MR imaging of the most commonly injured ankle ligaments. Part II. Ligament injuries. Radiology.

[18] Margetić P, Pavić R (2012). Comparative assessment of the acute ankle injury by ultrasound and magnetic resonance. Coll Antropol.

[19] Gün C, Unlüer EE, Vandenberk N, Karagöz A, Sentürk GO, Oyar O (2013). Bedside ultrasonography by emergency physicians for anterior talofibular ligament injury. J Emerg Trauma Shock.

[20] Cha SW, Bae KJ, Chai JW, Park J, Choi Y-H, Kim DH (2019). Reliable measurements of physiologic ankle syndesmosis widening using dynamic 3D ultrasonography: a preliminary study. Ultrasonography.

[21] Van Niekerk C, Van Dyk B (2017) Dynamic ultrasound evaluation of the syndesmosis ligamentous complex and clear space in acute ankle injury, compared to magnetic resonance imaging and surgical findings. S Afr J Radiol 21. 10.4102/sajr.v21i1.1191

[22] Mei-Dan O, Carmont M, Laver L (2013). Standardization of the functional syndesmosis widening by dynamic U.S examination. BMC Sports Sci Med Rehabil.

[23] Czyrny Z (2017). Standards for musculoskeletal ultrasound. J Ultrason.

[24] Calder JD, Bamford R, Petrie A, McCollum GA (2016). Stable versus unstable grade II high ankle sprains: a prospective study predicting the need for surgical stabilization and time to return to sports. Arthroscopy.

