# Supplementary material for: Diagnostic value of ultrasonography in acute lateral and syndesmotic ligamentous ankle injuries
Source: Eur Radiol. 2020 Oct 7;31(4):2610–20. doi: 10.1007/s00330-020-07305-7 (PMC7979658; doi:10.1007/s00330-020-07305-7)
Supplement: Supplementary file 1 — (DOCX 2556 kb) [file 330_2020_7305_MOESM1_ESM.docx]

**Supplementary appendix 1A** Cross-reference of Schneck Grade I injury of the anterior talofibular ligament (ATFL)


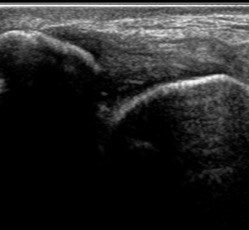
**
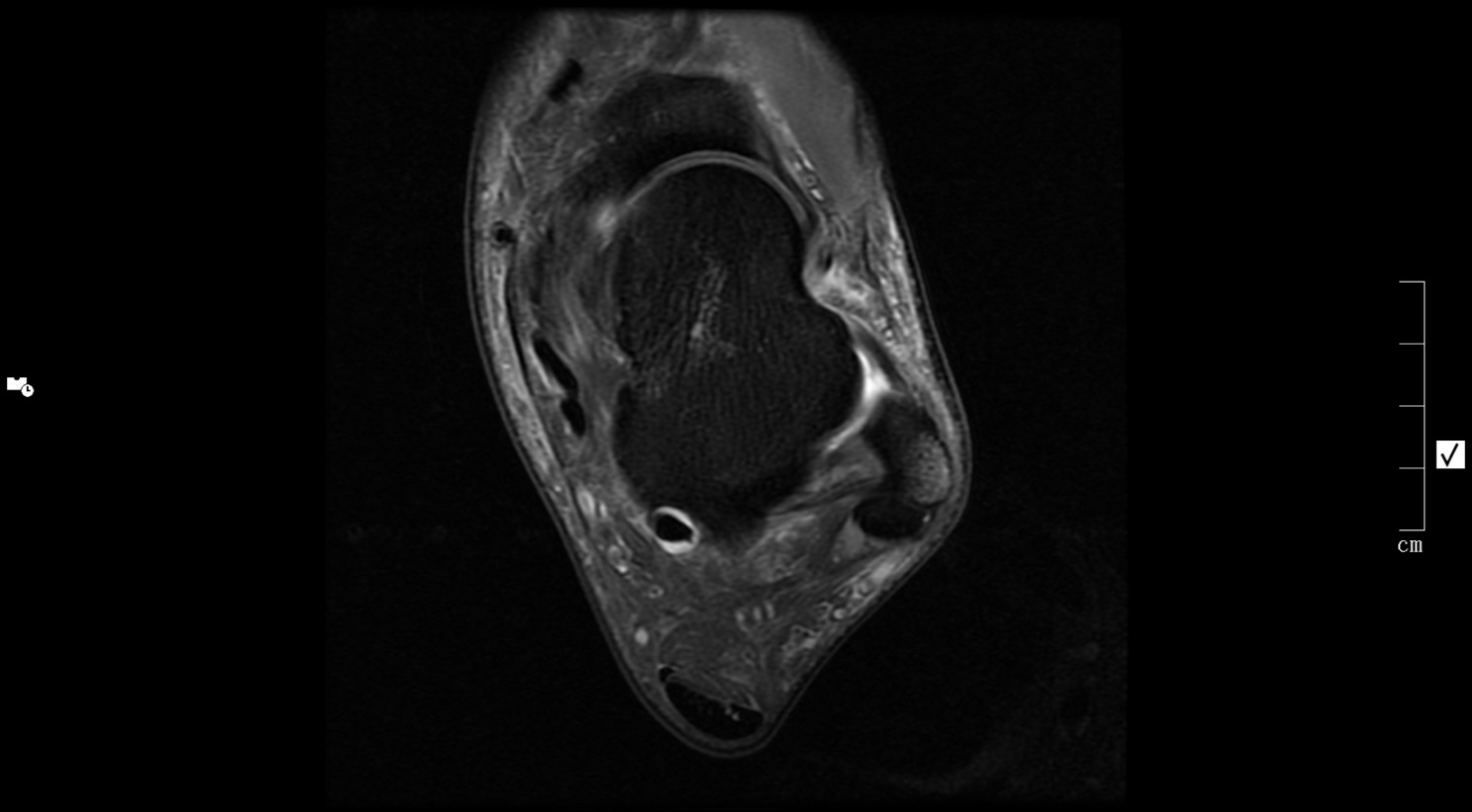

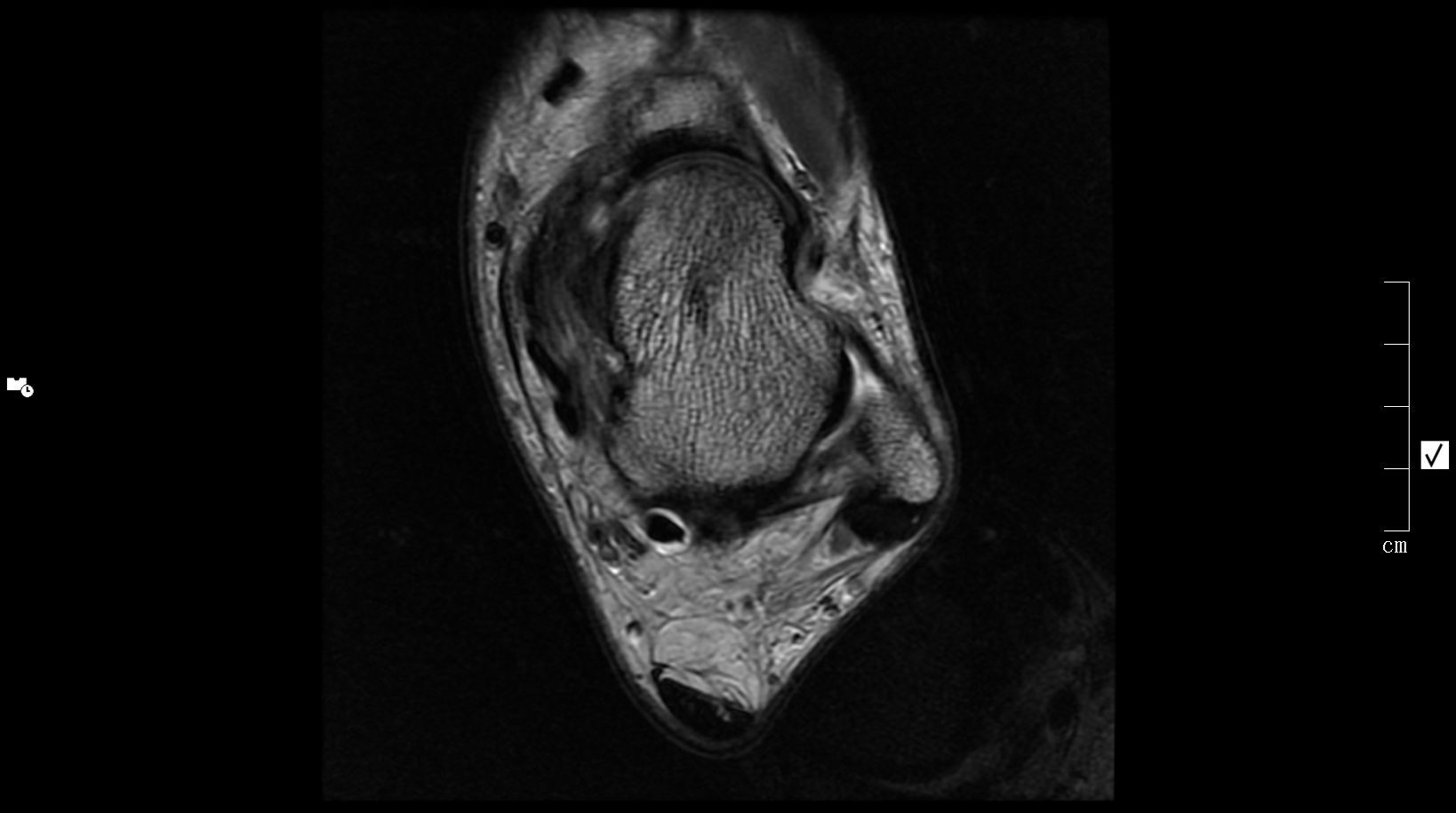
**

*

*

*

Cross-reference of Schneck grade I injury of the anterior talofibular ligament (ATFL) on Ultrasound and MRI; (**A**) Ultrasound image showing intact ATFL fibers (Asterisk) with peri-ligamentous hypoechoic halo consistent with soft tissue edema (Arrows) (**B**) Axial PD-FS image showing peri-ligamentous high signal/edema (Arrows) with intact fibers of the ATFL (Asterisk) (**C**) Axial T2-weighted image showing intact fibers of the ATFL (Asterisk).

**Supplementary appendix 1B** Cross-reference of Schneck Grade II injury of the anterior talofibular ligament (ATFL)


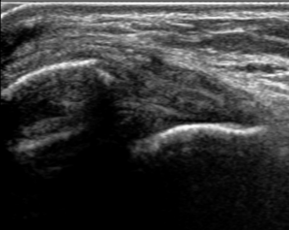


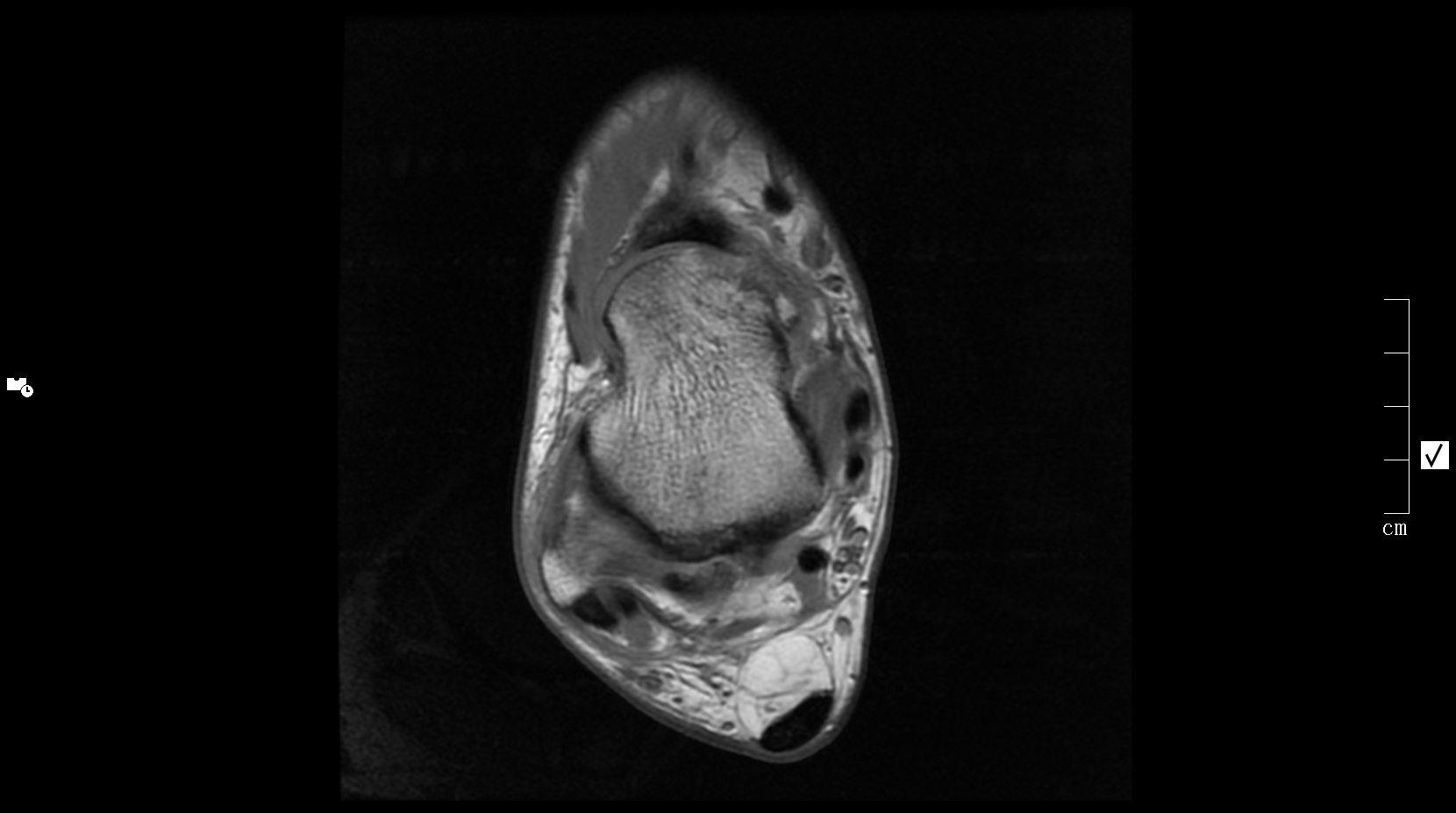


*


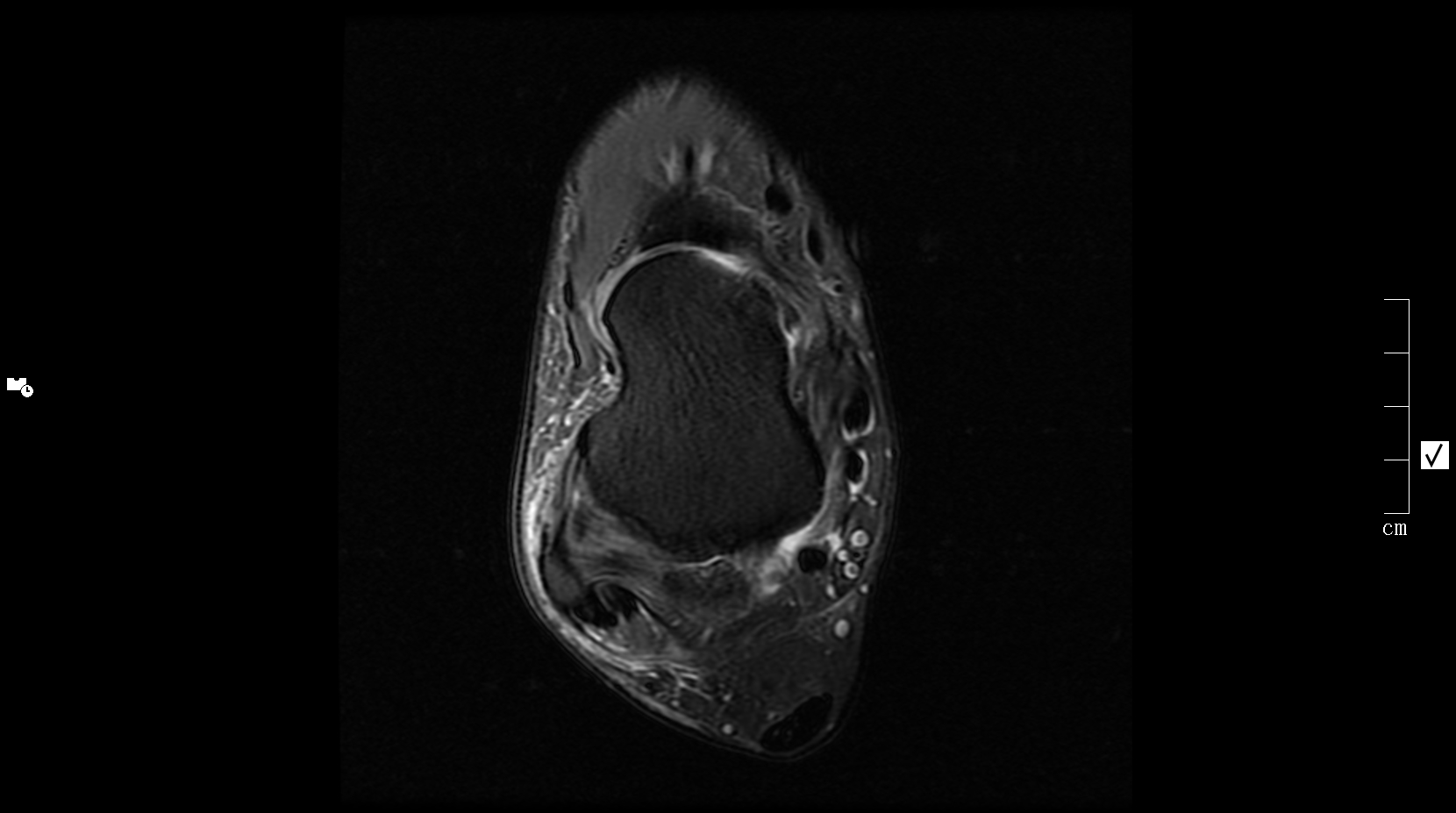


*

*

Cross-reference of Schneck grade II injury of the anterior talofibular ligament (ATFL) on Ultrasound and MRI; (**A**) Ultrasound image showing thickening and decreased echogenicity of the ATFL (Arrows) with partial discontinuity (Asterisk) (**B**) Axial PD-FS image showing a soft tissue edema surrounding the ATFL (Arrows) with some fibers of the ATFL remaining intact (Asterisk) (**C**) Axial T2-weighted image showing a partial tear of the ATFL (Arrows) with some fibers of the ATFL remaining intact (Asterisk)

**Supplementary appendix 1C** Cross-reference of Schneck Grade III injury of the anterior talofibular ligament (ATFL)


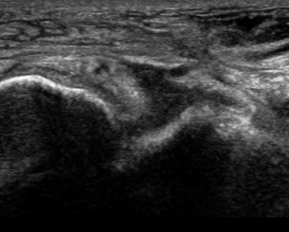


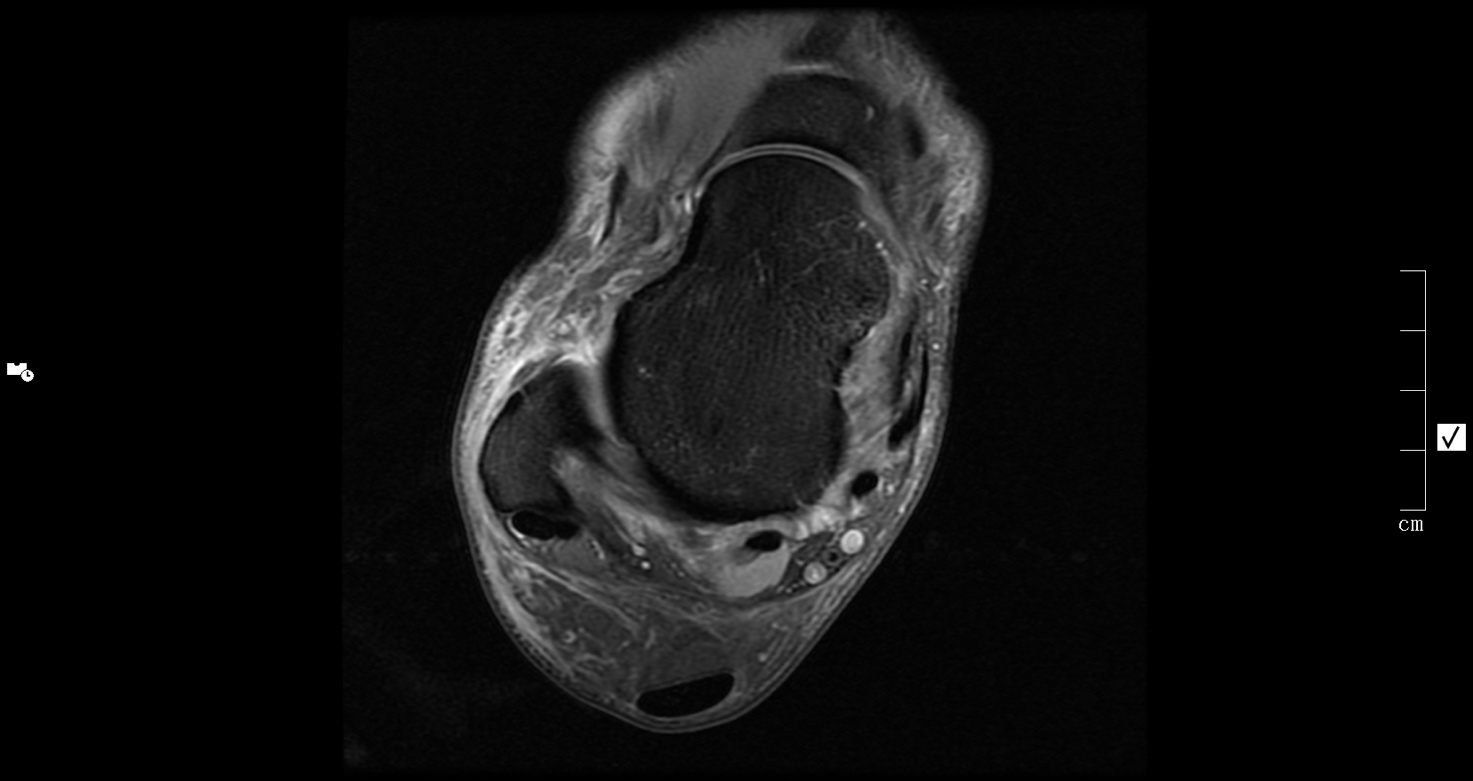

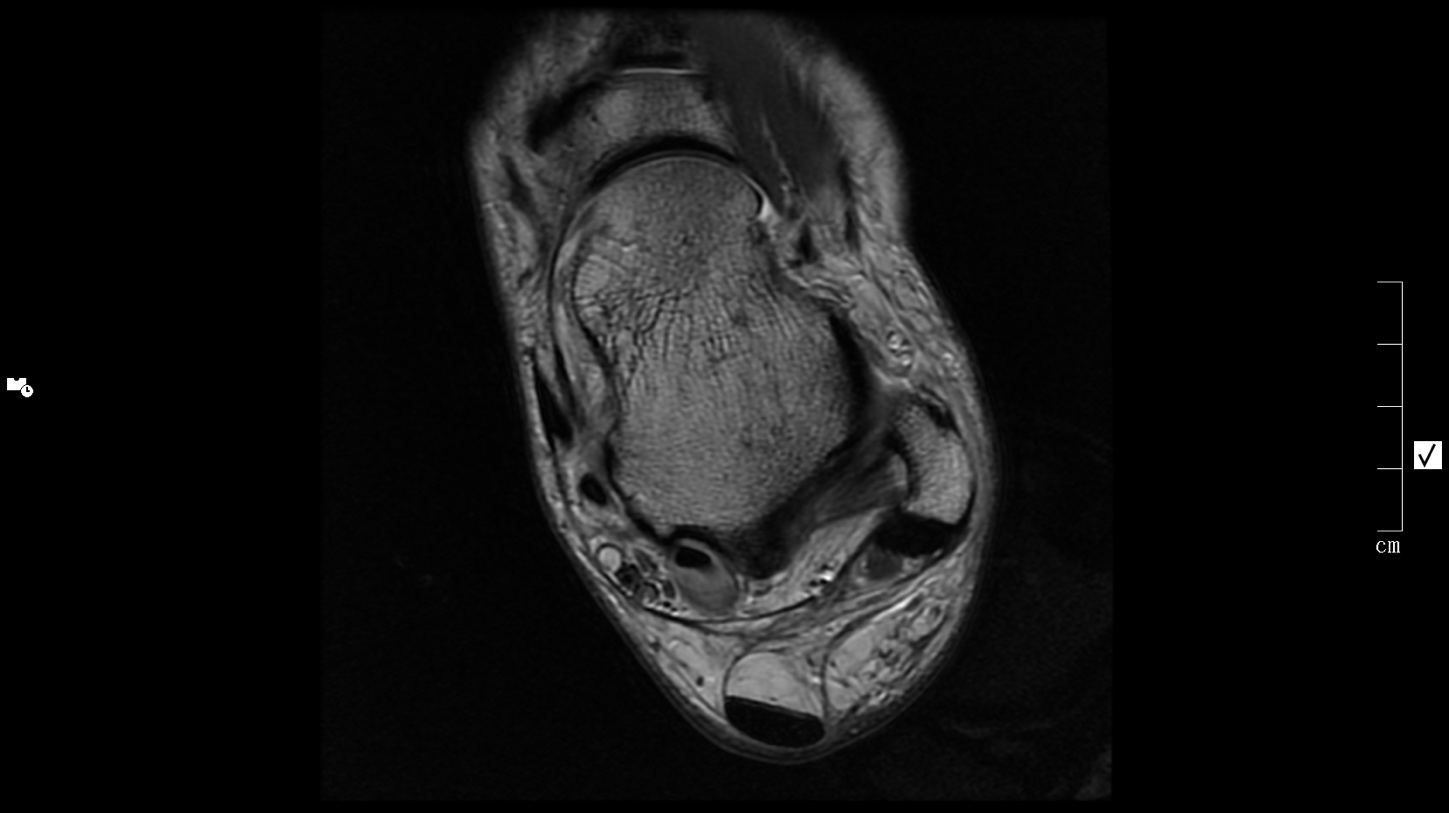


Cross-reference of Schneck grade III injury of the anterior talofibular ligament (ATFL) on Ultrasound and MRI (**A**) Ultrasound image showing a complete discontinuity of the ATFL (Arrows) (**B**) Axial PD-FS image showing soft tissue edema and complete ATFL discontinuity (**C**) Axial T2-weighted image showing no ATFL fibers remaining.

**Supplementary appendix 2:** Cross-tabulation for the grading of individual ankle ligaments according Ultrasound and MR imaging.

| *Lateral ankle ligaments* | | | | | |
| --- | --- | --- | --- | --- | --- |
| **ATFL** |  | **Grading per MRI** | | | |
|  | **Grading per US** | Normal | Grade 1 | Grade 2 | Grade 3 |
|  | Normal | 20 | 7 | 1 | 5 |
|  | Grade 1 | 0 | 0 | 1 | 0 |
|  | Grade 2 | 0 | 0 | 2 | 1 |
|  | Grade 3 | 3 | 1 | 10 | 41 |
| **CFL** |  | **Grading per MRI** | | | |
|  | **Grading per US** | Normal | Grade 1 | Grade 2 | Grade 3 |
|  | Normal | 37 | 6 | 16 | 7 |
|  | Grade 1 | 0 | 0 | 0 | 0 |
|  | Grade 2 | 0 | 1 | 9 | 5 |
|  | Grade 3 | 2 | 1 | 3 | 5 |
| *Syndesmosis ligaments* | | | | | |
| **AITFL** |  | **Grading per MRI** | | | |
|  | **Grading per US** | Normal | Grade 1 | Grade 2 | Grade 3 |
|  | Normal | 74 | 4 | 2 | 0 |
|  | Grade 1 | 0 | 0 | 0 | 0 |
|  | Grade 2 | 0 | 0 | 0 | 0 |
|  | Grade 3 | 0 | 0 | 0 | 12 |

Cross-tabulation of the Schneck grading by the MSK-radiologist according Ultrasound and MR imaging is provided for the individual ankle ligaments; 1) For the lateral ankle ligaments; anterior talofibular ligament (ATFL) and calcaneofibular ligament (CFL) and 2) the Syndesmosis ligaments; anterior inferior tibiofibular ligament (AITFL)
